# Small Drainage Volumes of Pleural Effusions Are Associated with Complications in Critically Ill Patients: A Retrospective Analysis

**DOI:** 10.3390/jcm10112453

**Published:** 2021-06-01

**Authors:** Benedikt Treml, Sasa Rajsic, Felix Diwo, Tobias Hell, Christoph Hochhold

**Affiliations:** 1 General and Surgical Intensive Care Unit, Department of Anaesthesiology and Critical Care Medicine, Medical University Innsbruck, 6020 Innsbruck, Austria; benedikt.treml@tirol-kliniken.at (B.T.); felix.diwo@student.i-med.ac.at (F.D.); 2 Department of Mathematics, Faculty of Mathematics, Computer Science and Physics, University of Innsbruck, 6020 Innsbruck, Austria; tobias.hell@uibk.ac.at

**Keywords:** chest tubes, critically ill, pleural effusion, pleural diseases, pneumothorax, small bore chest drains

## Abstract

Pleural effusions are a common finding in critically ill patients and small bore chest drains (SBCD) are proven to be efficient for pleural drainage. The data on the potential benefits and risks of drainage remains controversial. We aimed to determine the cut-off volume for complications, to investigate the impact of pleural drainage and drained volume on clinically relevant outcomes. Medical records of all critically ill patients undergoing insertion of SBCD were retrospectively examined. We screened 13,003 chest radiographs and included 396 SBCD cases in the final analysis. SBCD drained on average 900 mL, with less amount in patients with complications (*p* = 0.003). A drainage volume of 975 mL in 24 h represented the optimal threshold for complications. Pneumothorax was the most frequent complication (4.5%), followed by bleeding (0.8%). Female and lighter-weighted patients experienced a higher risk for any complication. We observed an improvement in the arterial partial pressure of oxygen and respiratory quotient (*p* < 0.001). We conclude that the small drainage volumes are associated with complications in critically ill patients—the more you drain, the safer the procedure gets. The use of SBCD is a safe and efficient procedure, further investigations regarding the higher rate of complications in female and lighter-weighted patients are desirable.

## 1. Introduction

Pleural effusions in critically ill patients are a common finding with an estimated annual incidence of more than 1.5 million in the United States (US) [1]. The most prevalent causes are volume overload, congestive heart failure, pleuro-pulmonary infections, atelectasis and cardiothoracic or major abdominal surgery [2,3,4,5,6,7,8,9,10]. Based on the method of detection, either using chest radiography or thoracic ultrasound, respectively, the described incidence of pleura effusion in critically ill patients ranges from 7.7% [11] to 62% [10].

Pleural effusions affect ventilation mainly by increasing the total thoracic volume and forcing the inspiratory muscles in a mechanically disadvantageous portion of their length-tension curve [12,13]. Furthermore, gas exchange is impaired by a mildly increased fraction of shunt, being the main mechanism for consecutive arterial hypoxemia [14].

From a clinical point of view, the risk-benefit-ratio of pleural drainage in the critically ill is still unknown. Procedures are generally considered safe, the most common reported complications being pneumothorax, followed by hemothorax, inappropriate catheter positioning, infection and re-expansion pulmonary oedema [15,16]. Regarding the benefits, a recent study showed improved oxygenation after drainage within 24 h of detection of pleural effusions in 76 intensive care unit (ICU) patients. In the second group with expectant management in 150 patients oxygenation remained unchanged [17]. An US study demonstrated a decrease of about 5% of shunt after drainage of pleural effusion in 22 ICU patients [18]. Other mechanisms of improvement may include respiratory mechanics itself. Nearly four decades ago, Estenne et al. demonstrated a decreased pleural pressure, thus increasing end-expiratory transpulmonary pressure [12]. Furthermore, increased dynamic compliance, respiratory system resistance, and end-expiratory lung volume have been demonstrated [15,19,20,21]. As a consequence, drainage of effusions may exert beneficial effects on weaning from mechanical ventilation or even allow it [22].

However, simple oxygenation does not entirely determine clinical outcome. A few small prospective studies sought to clarify an association between impaired oxygenation and clinical outcome parameters. Over two decades ago, Mattison et al. observed longer ICU stays and longer duration of mechanical ventilation in 100 ICU patients with pleural effusions [10]. More recently, a French multicentre trial observed a higher rate of failed spontaneous breathing trials, fewer ventilator-free days at 1 month, and a nearly doubled ICU mortality in 249 patients with moderate-to-large pleural effusion [23]. This is in line with a retrospective analysis which found pleural effusions in nearly 4000 critically ill patients to be associated with increased mortality, draining these effusions increased mortality even more [11]. On the other hand, drainage may aid in establishing a diagnosis and thus leading to therapy [24]. The most recent meta-analysis was inconclusive regarding the overall impact on relevant clinical outcomes, these large studies revealed more questions than answers.

To contribute valuable information, we sought to identify possible risk factors for the prediction of complications in pleural drainage in critically ill patients.

## 2. Materials and Methods

### 2.1. Patient Selection

We retrospectively reviewed all the patients admitted to the General and Surgical ICU between 1 January 2016 and 31 December 2018. This tertiary ICU of the Department of Anaesthesia and Critical Care Medicine, Medical University Innsbruck, Austria treats surgical, polytraumatized, as well as medical patients.

All critically ill patients undergoing pleural puncture with consequent insertion of a ten French small bore chest drain (SBCD) (Navarre Opti-Drain, Bard Access Systems, Becton Dickinson GmbH, Heidelberg, Germany) were enrolled. Multiple drainage procedures in one patient were considered as separate cases as the risk of complication is present within every procedure. Therefore, we report our results for both all included patients and per patient case.

In general, all the patients at our ICU are screened for pleural effusions on a daily basis using a chest radiograph. After sonographic confirmation of an inter-pleural separation of more than 2 cm, the indication for drainage is established by the treating clinicians after a meticulous risk assessment. The ultrasound guided drainage procedure follows international guidelines [25]. All the procedures were followed by plain chest radiography within 3 and 24 h.

Two authors (F.D. and B.T.) checked independently each radiograph and the corresponding radiology report for SBCD, as well as potential complications. All the pleural drains were placed by experienced residents or consultants for anaesthesia and intensive care medicine.

Exclusion criteria were the placement of SBCD prior to ICU admission, drainage of cavities other than the pleural cavity, insertion by other than the treating clinicians, and an age less than 18, respectively.

We obtained (1) socio-demographic data including age, sex, body weight and height, body mass index, as well as simplified acute physiology score 3 (SAPS III) [26]; (2) date and time of insertion of the small bore chest drain, drainage volume in 24 h, anteroposterior chest radiography and radiology report; (3) blood gas analysis including arterial partial pressure of oxygen (paO_2_), arterial partial pressure of carbon dioxide, haemoglobin and sodium concentration directly before and 30 min after the intervention; (4) the fraction of inspired oxygen and the respiratory quotient; (5) coagulation status including platelets count, prothrombin time (PT), activated partial thromboplastin time (aPTT), and fibrinogen; and (6) complications. Reported complications comprised of pneumothorax, severe bleeding, inappropriate positioning, and re-expansion pulmonary oedema.

Complications were considered as being severe when they required subsequent intervention, such as placement of other drainages, surgical or angiographic therapy, additional administration of blood products or coagulation therapy. A simple misplacement without further implications was considered severe in the case of organ perforation, intra-abdominal or intra-spinal drain position.

This retrospective study was approved by the Ethics Committee of the Medical University of Innsbruck, Austria (Ethic-Committee-Number: 1014/2018).

### 2.2. Outcomes

The primary objective of this study was to investigate an association of complications with the volume of drained pleural effusion. Secondary outcomes included drainage volume in the first 24 h, overall complication rate, change in oxygenation and ventilation, ICU mortality, in-hospital mortality, and change in coagulation parameters.

### 2.3. Statistical Analyses

A mathematician not involved in study procedures or patient assessment performed the statistical analyses using R, version 3.4.2 (R—free software for statistical computing and graphics, R Foundation for Statistical Computing, Vienna, Austria). All statistical assessments were two-sided and a significance level of 5% was used. The Wilcoxon rank sum test and Fisher’s exact test were applied to assess differences between intention-to-treat (ITT) groups. We present continuous data as medians (25th–75th percentile) and binary variables as no./total no. (%). We show the effect size and precision with estimated median differences for continuous data and odds ratios (OR) for binary variables, with 95% confidence intervals (CI).

For complications associated with the drainage amount in 24 h, a ROC analysis was performed and the resulting ROC curve is shown. Similar to the effect size, we provide the area under the ROC curve (AUC) with a 95% CI. In addition, the optimal threshold for the drainage amount due to Youden’s index was computed.

## 3. Results

### 3.1. Study Population

**Figure 1:**
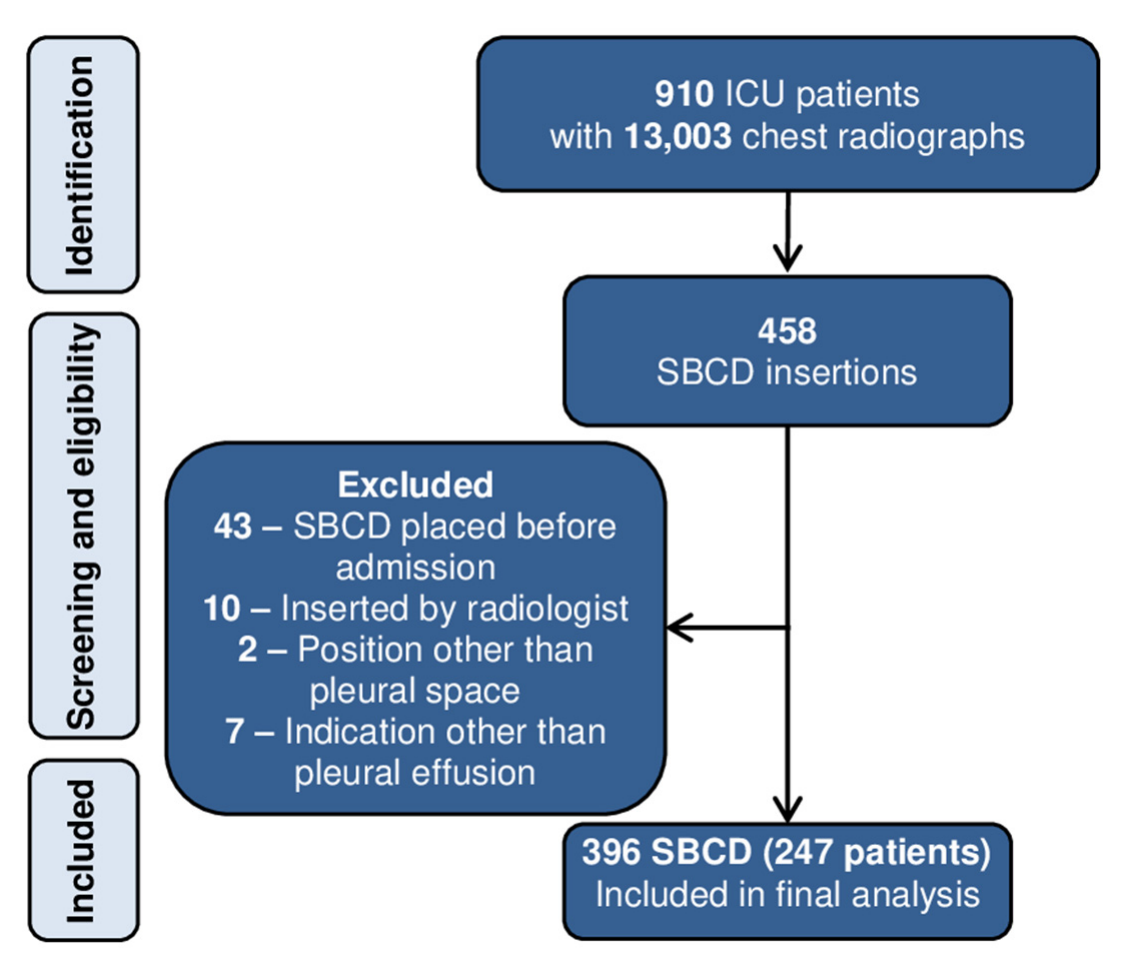
Screening and eligibility flow chart. ICU: Intensive care unit; SBCD: Small bore chest drain.

Over a period of 3 years, 910 patients were admitted to our ICU. After screening of 13,003 chest radiographs, 458 SBCD were identified. Finally, our study population comprised of 247 patients with 396 inserted SBCD for drainage of pleural effusion (Figure 1).

Demographics of patients are reported in Table 1. Patients with pleural effusions drainage were predominantly male, with a median age of 71 and a SAPS III score of 64 points upon admission.

Within 396 procedures complications occurred in 22 (5.6%) cases. Patients with complications weighed less than those without complications. Moreover, female patients had more complications (Table 1). When assessing all SBCD cases, female sex showed only a trend towards more complications without reaching significance (data not shown). Baseline characteristics of the patient population per case are shown in Table 2.

### 3.2. Drainage Volume

**Figure 2:**
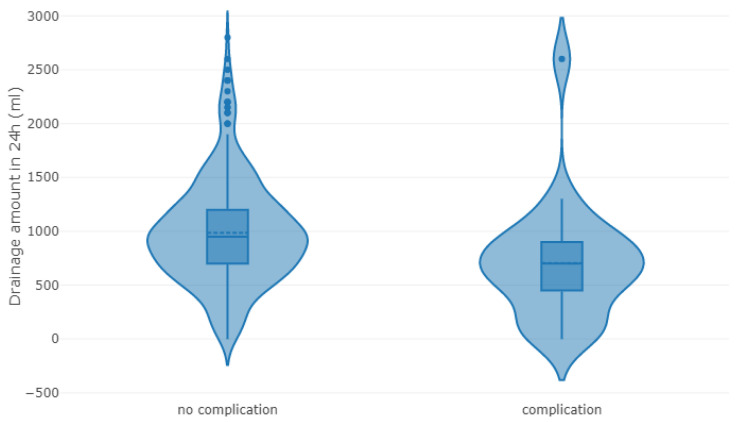
Plots of drainage volume in 24 h (mL) for patients without and with complications.

The average drainage volume was 900 mL in the first 24 h. In patients without complications, SBCD drainage volume was significantly higher than in patients with complications (950 vs. 700 mL, *p* = 0.003), see Table 3 and Figure 2.

**Figure 3:**
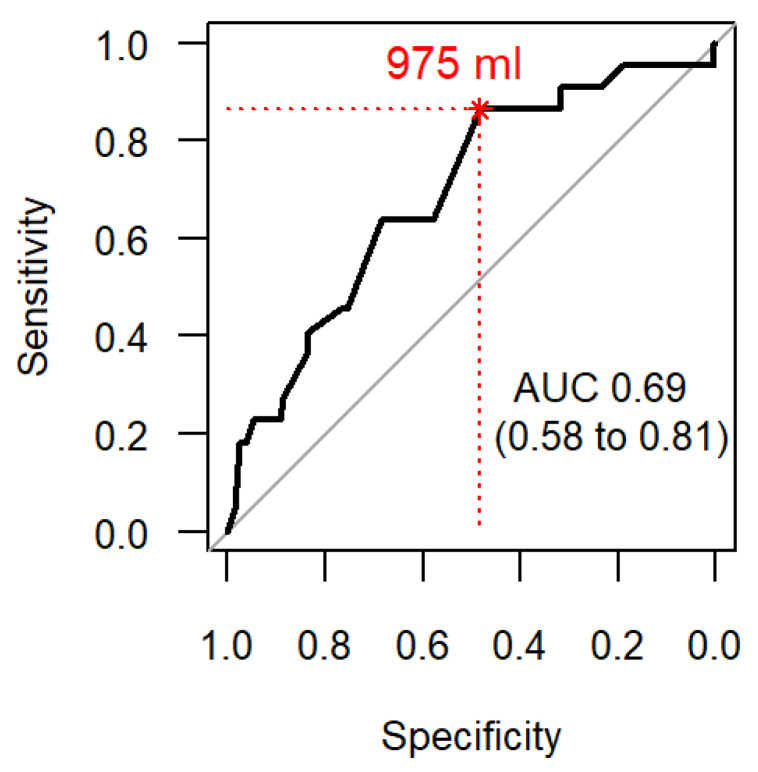
The receiver operating characteristic (ROC) curve for complications associated with drainage volume in 24 h (mL). The optimal threshold due to Youden’s index for the drainage volume (red number) corresponds to the point closest to the top-left marked with an asterisk. AUC: Area under the ROC curve.

When calculating the discrimination threshold for complications, the receiver operating characteristic (ROC) curve showed that a drainage volume of 975 mL in 24 h represented the optimal threshold for the association with complications, see Figure 3.

### 3.3. Complications

Over a 3-year period, the complication rate decreased from 8.1% in 2016, to 7.6% in 2017, and 6.1% in 2018, respectively. Within the group of critically ill patients who developed complications in the first 24 h, 18 (81.9%) experienced pneumothorax, three (13.7%) bleeding events, and one (4.6%) an epidural malposition. Six out of 18 pneumothoraces were classified as severe, requiring additional treatment and chest drainage. Moreover, all pneumothoraces were associated with the SBCD insertion. Two of three bleeding events arose from a pulmonary artery, and one from an intercostal artery, all requiring urgent angiographic intervention. Finally, in one case, the SBCD insertion occurred through a intervertebral foramen into the subarachnoid space, resulting in liquor drainage without any neurological sequelae. Due to the high risk of adverse effects, this malposition was ruled as being severe despite the fact that no further interventions were necessary. Out of the total 22 that observed complications, ten were classified as severe and twelve as mild. None of the patients experienced re-expansion pulmonary oedema.

### 3.4. Oxygenation

After drainage, the fraction of inspired oxygen was reduced, paO_2_ increased, and the respiratory quotient improved by 18%. Other clinical parameters remained unchanged (Table 3).

### 3.5. Mortality

No patients died in the direct relation to the pleural effusion drainage procedures. From 247 patients, 55 patients died in the hospital, from which 41 died already at the ICU (with a median ICU survival time of 26 days), see Table 4. The calculated SAPS III was 64 points with an observed ICU mortality of 16.6%. The ICU-and in-hospital mortality were higher in the group without complications.

## 4. Discussion

This retrospective single-centre study demonstrated a higher risk for complications after drainage of small volume pleural effusions. We could demonstrate a drainage volume of 975 mL in 24 h after pleural puncture being the optimal threshold for complications in critically ill patients. Interestingly, female sex represented more than half of the patients with complications. This could be partly explained through a lower body-mass index (BMI) and weight, as adult females in Austria show low BMI values in all BMI categories [27]. Ault et al. demonstrated a nearly 3-fold higher rate of pneumothorax in underweight patients in nearly 10,000 thoracocentesis procedures [28], while Cho et al. demonstrated significantly more pneumothoraces in patients with lower BMI [29]. Compared to the literature, our critically ill patients without complications were in the lower range of overweight (BMI 25.1; data not shown). Underlying mechanisms may be shorter distances between chest wall and pleura or a smaller intercostal space with consequently lesser space for the adjoining blood vessels. However, we observed an equally distributed height in both groups.

### 4.1. Drainage Volume

At our ICU, the established SBCD drained an average 969 mL of pleural fluid in the first 24 h. Previous observations correspondingly demonstrated about 1000 mL of drained fluid within the first 24 h [22,30,31].

In our critically ill patients experiencing complications, the average drainage volume was 250 mL lower compared to those without complications. Moreover, a smaller drainage volume has been shown to be associated with a higher rate of complications. Therefore, using the calculated threshold of 975 mL as an additional criterion could identify patients at higher risk for complications.

The amount of liquid in pleural space can be predicted using the Goecke and Schwerk formula published for the first time in 1990 [32]. In this formula, the maximal craniocaudal distance of effusion in centimeters is added to the height of the subpulmonal effusion and multiplied by factor 70, resulting in the anticipated volume of pleural effusion in milliliters, having the highest reported accuracy. Few other equations were later proposed, but with a lower accuracy [33]. According to the Goecke and Schwerk equation, and using our threshold, the decision on pleural drainage procedure should be carefully considered if the sum of craniocaudal and subpulmonal distances is less than 14 cm (14 × 70 = 980 mL).

As early as 1993, Bartter et al. reported pneumothorax as a complication in patients with smaller pleural effusions. However, due to the small number of subjects they could neither specify the volume nor report any correlation between the size of effusion and pneumothorax [34]. Data from the middle nineties showed no association between the drained volume and pneumothorax rate in 44 patients requiring intensive care [6,35]. In light of this gathered data, our study is—to the best of our knowledge—one of the largest reporting on an association of drainage volume and complications in critically ill patients.

### 4.2. Complications

The overall rate of complications in critically ill patients was 5.6% for the observed period of 3 years, with a tendency of reduction throughout the years, most probably due to the improved practice of ultrasound usage over time. The most common complication within the first 24 h was pneumothorax (4.6%), which is in line with a 10-year old systematic review observing 4.3% in 496 ICU patients [31]. Recently, Vetrugno et al. observed 21.8% of pneumothoraces, all being small (maximum 20 mm) and without clinical significance in 71 critically ill patients. This high rate has been explained by the low experience of residents using an ultrasound [36]. Changing levels of experience and routine are the established risk factors for complications in pleural puncture [34,37,38]. Surprisingly, in the mentioned Canadian review, the use of ultrasound during thoracocentesis in mechanically ventilated patients was not associated with a reduction in pneumothorax (OR 0.32, 95% CI, 0.08% to 1.19%). In other prospective studies, the pneumothorax incidence was around or below 5% [17,39,40,41,42] with some higher rates in ICU-settings [31]. Recently, two large studies reported an incidence of pneumothoraces below 1% but none of them included critically ill patients in an ICU-setting [28,29]. In a review by Gordon et al., one third of pneumothoraces were classified as severe and required further surgical treatment [37]. This is comparable to our findings with six out of 18 pneumothoraces being ruled as severe.

Most recently, a prospective Australian study reported a higher rate of complications ranging from 10.5% when drainage occurred within 24 h and up to 16% with expectant management in 226 ICU patients [17]. Counting only pneumothoraces in the group with early drainage, the complication rate was half to 5.3% (four out of 76), being consistent with our findings. Given the progress of modern ultrasound technology and other imaging techniques, complication rates ranging from 20% to as high as 46% before the 1990s are not surprising [43,44].

In regards to hemothorax, Goligher et al. reported a risk of 1.6% (95 CI, 0.8% to 3.3%) from 10 studies with 721 patients [15]. Two retrospective studies included in this review demonstrated a risk for bleeding events of 1.4% and 3.7%, respectively [30,31]. This is twice as much compared to our data with 0.8% of severe bleeding events leading to hemothorax. However, the low number of bleeding events in our work needs cautious interpretation.

Other reported complications are inappropriate positioning of SBCD in 1.3% [36,42] and kinking of the catheter in 6.5% of patients [30]. In our cohort, one severe complication with malposition into the epidural space occurred (0.3%). We did not observe other rare complications such as subcutaneous hematomas, re-expansion pulmonary oedema or empyema [30] in our study.

### 4.3. Oxygenation

We demonstrated an improvement in oxygenation with an increase of the respiratory quotient, being in line with a meta-analysis calculating an 18% improvement in 118 mechanically ventilated patients from five studies [15]. However, due to the different time points of blood gas measurements, the underlying studies showed heterogeneous results. Two of them (with 31 patients) reported non-significant and minor increases within 1 h after thoracocentesis [18,45]. Similar results have been shown if drawing blood gases after 12 h (*n* = 22, [22]) or after 24 h (*n* = 24, [31]). Two studies showed a distinct increase in 43 patients [20,46]. However, in the work from Roch et al., half of the patients with small size effusion showed an unchanged respiratory quotient [46].

Fysh et al. compared ICU patients with pleural effusion either receiving early SBCD within 1 day or an expectant strategy including less and later drainage procedures. This Australian group found a marked improvement of the respiratory quotient only in the first group [17]. Moreover, they demonstrated a similar rate of complications in both groups [17]. Roch et al. and Razazi et al. reported improved oxygenation if draining pleural effusion with volumes greater than 500 mL [21,46]. This is compatible with work from the UK observing an increase in the paO_2_ of 4 mmHg for every 100 mL of drained effusion [47]. The appliance of this formula to our patients should have led to a greater increase of paO_2_, with nearly a litre of drained effusion.

Regarding the improvement in oxygenation, several mechanisms have been discussed: reduced end-expiratory pleural and transpulmonary pressures, an increased lung volume, and increased dynamic compliance [12,15,19,20,21]. In regards to pulmonary perfusion-ventilation distribution, conflicting results have been observed. While in a small cohort of seated patients an increased pulmonary shunt fraction after drainage has been shown [14], exactly the opposite effect was demonstrated in ICU-patients [18]. On the other hand, Walden et al. revealed an increased fraction of dead space in 10 critically ill patients with pleural effusions with a small reduction of dead space after 24 h of drainage [47]. In summary, these various findings on oxygenation and respiratory mechanics may reflect the variety of underlying aetiologies and different (respiratory) therapies raising the question of impact on mortality.

### 4.4. Mortality

In this retrospective study, none of the patients died in direct relation to the pleural drainage procedures, which is consistent with the literature. We observed an ICU mortality of around 17% being lower than predicted by SAPS (median SAPS III score of 64), and 22% in-hospital mortality, respectively. Patients without complications showed a higher mortality. Given the low number of deaths in the complications group this needs to be interpreted cautiously. The outcome in our study is slightly better than the median value reported in a recent European multicentre cohort study with an overall ICU mortality of 19.5% but distinct lower median SAPS III scores of 35 [48]. In 2018, Razazi et al. observed an ICU mortality of 16% in 81 patients with moderate-to-large pleural effusion at their first spontaneous breathing trial [23]. However, in this study, the reported median SAPS II scores (median 52) were also lower. The before mentioned Australian prospective trial showed no effect of early drainage versus expectant management on mortality, length of ICU stay or duration until extubation [17]. Recently, the largest retrospective analysis of more than 50,000 ICU patients reported an in-hospital mortality as high as 35% in 3897 patients with pleural effusions. Furthermore, mortality increased to 43.9% after pleural drainage in 1503 patients [11].

### 4.5. Limitations

Selecting patients for pleural drainage was based upon a meticulous risk-benefit analysis by the treating clinicians. However, the selection bias cannot be excluded. Moreover, the patient population at our intensive care unit could differ from other ICUs. As we focused on the identification of risk factors of pleural drainage we did not report the underlying causes. Relying on chest radiographs alone may lead to missing an even large volume of pleural effusion. However, due to the extensive use of ultrasound the chance seems very little.

Since our objective was the outcome within 24 h, we did not report on potential infections and pneumothorax upon SBCD removal. Moreover, we did not assess pleural pressure. A pneumothorax may change the pleural pressure attenuating the drainage capability of a SBCD.

## 5. Conclusions

To the best of our knowledge, this is the first study to investigate the impact of drainage volume on clinically relevant outcomes in an European ICU setting. Moreover, we demonstrate a drainage volume of less than 975 mL being associated with complications in our sample of 396 inserted SBCD. Given the small rate of complications, the use of SBCD appears to be a safe and efficient procedure in an ICU setting. Drainage of pleural effusions is still no panacea but one measure in a broad bundle of acute respiratory care. Clearly, further research is warranted to clarify the underlying mechanisms of a higher risk for complications in female sex and lighter patients.

## Figures and Tables

**Table 1:** Demographics ^a^ of patient population (*n* = 247).

Baseline Characteristics	Total (*n* = 247)	No Complications (*n* = 225)	Complications (*n* = 22)	Estimate with 95% CI ^b^	*p*-Value ^c^
Age (years)	71	71	69	71 (65.54; 69.22)	0.519
Female sex	82 (33.2%)	70 (31.1%)	12 (54.5%)	2.65 (0.99; 7.19)	0.040
Height (cm)	173 (165–178)	174 (165–178)	171 (160–175)	3 (0; 7)	0.080
Weight (kg)	75 (65–85)	75 (67–85)	65 (55–79.5)	8.5 (1; 15.2)	0.028
SAPS III at admission (pts)	64 (55–74)	64 (55–74)	62.5 (52–70.7)	1.7 (−5; 9)	0.638

SAPS III: Simplified acute physiology score 3. ^a^ Binary data are presented as no./total no. (%), continuous data as medians (25th to 75th percentile). ^b^ Odds ratios for binary variables and estimated median difference for continuous variables. ^c^ Assessed by Fisher’s Exact test for categorical variables and Wilcoxon Rank Sum test for continuous variables.

**Table 2:** Baseline characteristics ^a^ per case (*n* = 396 SBCD).

Baseline Characteristics	Total (*n* = 396)	No Complications (*n* = 374)	Complications (*n* = 22)	Estimate with 95% CI ^b^	*p*-Value ^c^
**Oxygenation**					
FiO_2_ (%)	45 (30–55)	45 (30–55)	45 (31.3–50)	0 (−5; 5)	0.874
paCO_2_ (mmHg)	41.2 (36.6–45.5)	41.4 (36.7–45.6)	39.3 (35.8–40.9)	1.6 (−1.4; 4.3)	0.245
paO_2_ (mmHg)	83.6 (73.1–96.1)	83.4 (72.8–95.9)	86.8 (77.1–104.3)	−3.9 (−11.7; 4)	0.323
Respiratory quotient	195.2 (145.9–269.8)	195.2 (145.6–269.4)	197.1 (154.0–296.9)	−11.9 (−50.0; 26.8)	0.539
**Coagulation**					
Haemoglobin (g/dL)	8.9 (8.3–9.5)	8.9 (8.3–9.5)	9 (8.43–9.3)	−0.1 (−0.5; 0.3)	0.688
Sodium (mmol/L)	142 (139–144)	142 (139–144)	140.5 (138.3–143.0)	1 (−1; 3)	0.246
Platelets (G/L)	153.5 (100.0–232.8)	153 (97.8–232.3)	171.5 (112.8–265.5)	−19 (−63; 23)	0.345
Prothrombin index (%)	73 (61–81)	73 (61–81)	69 (59.3–92.5)	0 (−10; 8)	0.970
aPTT (s)	42 (37–48)	42 (37–48.25)	41 (36.3–47.3)	2 (−2; 5)	0.423
Fibrinogen (mg/dL)	437 (333.5–532.8)	437 (333.0–532.3)	439.5 (355–545)	−7 (−75; 59)	0.828

FiO_2_: Fraction of inspired oxygen; paCO_2_: Arterial partial pressure of carbon dioxide; paO_2_: Arterial partial pressure of oxygen; aPTT: Activated partial thromboplastin time. ^a^ Binary data are presented as no./total no. (%), continuous data as medians (25th to 75th percentile). ^b^ Odds ratios for binary variables and estimated median difference for continuous variables. ^c^ Assessed by Fisher’s Exact test for categorical variables and Wilcoxon Rank Sum test for continuous variables.

**Table 3:** Outcome parameters ^a^ per case (*n* = 396 SBCD).

Outcome Parameters	Total (*n* = 396 SBCD)	No Complications (*n* = 374 SBCD)	Complications (*n* = 22)	Estimate with 95% CI ^b^	*p*-Value ^c^
Drainage volume in 24 h	900 (650–1200)	950 (700–1200)	700 (462.5–900)	300 (100; 500)	0.003
**Change in oxygenation after SBCD insertion**					
FiO_2_ (%)	0 (0–0)	0 (0–0)	0 (0–0)	0 (0–0)	0.398
paCO_2_ (mmHg)	0.45 (−1.9–3.0)	0.45 (−1.9–2.9)	0.25 (−1.3–4.4)	−0.1 (−2.2; 1.8)	0.874
paO_2_ (mmHg)	8.7 (−8–23)	9 (−7.2–22.6)	2.8 (−11.5–25.9)	4.1 (−7.9; 14.6)	0.495
Respiratory quotient	20 (−15.3–56.0)	20.7 (−15–56)	8.5 (−25.8–44.3)	11.8 (−14.8; 37.4)	0.372
**Change in coagulation parameters after SBCD insertion**					
Hemoglobin (g/dL)	0 (−0.3–0.2)	−0.1 (−0.3–0.2)	0.1 (−0.5–0.2)	0 (−0.2; 0.3)	0.602
Sodium (mmol/L)	0 (−1–1)	0 (−1–1)	0 (−1–2)	0 (−1; 1)	0.778
Platelets (g/L)	4 (−9–17)	3 (−8.5–16)	10.5 (−20.8–29.5)	−5 (−20; 11)	0.514
Prothrombin index (%)	0 (−5–4)	0 (−5–4)	0.5 (−8.5–8.5)	0 (−5; 4)	0.884
aPTT (s)	0 (−2–2)	0 (−2–3)	1 (−2–2)	0 (−2; 1)	0.794
Fibrinogen (mg/dL)	1 (−29.8–30.8)	0 (−30–30)	16 (−20–36)	−11 (−34; 11)	0.306

SBCD: Small bore chest drains; FiO_2_: Fraction of inspired oxygen; paCO_2_: Arterial partial pressure of carbon dioxide; paO_2_: Arterial partial pressure of oxygen; aPTT: Activated partial thromboplastin time. ^a^ Binary data are presented as no./total no. (%), continuous data as medians (25th to 75th percentile). ^b^ Odds ratios for binary variables and estimated median difference for continuous variables. ^c^ Assessed by Fisher’s Exact test for categorical variables and Wilcoxon Rank Sum test for continuous variables.

**Table 4:** Mortality ^a^ of study patients (*n* = 247).

Outcome Parameters	Total (*n* = 247)	No Complications (*n* = 225)	Complications (*n* = 22)	Estimate with 95% CI ^b^	*p*-Value ^c^
ICU mortality	41 (16.6%)	39 (17.3%)	2 (9.1%)	0.48 (0.05; 2.1)	0.547
In-hospital mortality	55(22.3%)	52 (23.1%)	3 (13.6%)	0.53 (0.1; 1.89)	0.424

ICU: Intensive care unit. ^a^ Binary data are presented as no./total no. (%), continuous data as medians (25th to 75th percentile). ^b^ Odds ratios for binary variables and estimated median difference for continuous variables. ^c^ Assessed by Fisher’s Exact test for categorical variables and Wilcoxon Rank Sum test for continuous variables.

## Data Availability

The datasets used and analyzed during the current study are available from the corresponding author on reasonable request.
